# The Four Epochs of Woman’s Life

**Published:** 1904-01

**Authors:** 


THE FOUR EPOCHS OF WOMAN'S LIFE. Maidenhood, Marriage, Maternity, Menopause. By ANNA M. GALBRAITH, M. D., Author of Hygiene and Physical Culture for Women. With an introductory note by John H. Musser, M. D., Professor of Clinical Medicine, University of Pennsylvania. Duodecimo volume of 247 pages. Second edition, revised and greatly enlarged. Philadelphia, New York, London: W. B. Saunders & Company. 1903. (Cloth, $1.50 net.)

The difficult topic of sexual hygiene has been most admirably handled by this author and the compliment of an early second edition is a deserved one. The sections on hygiene of puberty, hemorrhage at the menopause, and hygiene of the menopause are new and supply what was lacking in the earlier edition. No better book exists of its kind; it is modest and, above all, sensible. Put into the hands of the expectant wife it cannot fail to conserve both her health and happiness and to make her a woman better fitted for motherhood. M. J. F.

